# Soldier-Health

**Published:** 1862-10

**Authors:** W. W. Hall

**Affiliations:** New-York


### SOLDIER-HEALTH.

BY DR. W. W. HALL, NEW-YORK,

*Editor of Hall's Journal of Health.*

---

1. In an ordinary campaign, sickness disables or destroys three times as many as the sword.

2. On a march, from April to November, the entire clothing should be a colored flannel shirt, with a loosely-buttoned collar, cotton drawers, woolen pantaloons, shoes and stockings, and a light-colored felt hat, with broad brim to protect the neck, eyes, and face from the glare of the sun and from the rain, and a substantial but not heavy coat when off duty.

3. Sun-stroke may be prevented by wearing a silk handkerchief in the hat, a few green leaves, a dampened sponge, or a white linen hood hat-cover, extending like a cape over the neck and shoulders.

4. Colored blankets are best, and if lined with brown drilling the warmth and durability are doubled, while the protection against dampness from lying on the ground, is almost complete.

5. Never lie or sit down on the grass or bare earth for a moment; rather use your hat—a handkerchief even, is a great protection. The warmer you are, the greater need for this precaution, as a damp vapor is immediately generated, to be absorbed by the clothing, and to cool you off too rapidly.

6. While marching, or on other active duty, the more thirsty you are, the more essential is it to safety of life itself, to rinse out the mouth two or three times, and *then* take a swallow of water at a time, with short intervals. A brave French general, on a forced march, fell dead on the instant, by drinking largely of cold water, when snow was on the ground.

7. Abundant sleep is essential to bodily efficiency, and to that alertness of mind which is all-important in an engagement; and few things more certainly and more effectually prevent sound sleep than eating heartily after sun-down, especially after a heavy march or desperate battle.

8. Nothing is more certain to secure endurance and capability of long-continued effort, than the avoidance of every thing as a drink except cold water, NOT excluding coffee at breakfast. Drink even cold water very slowly, and as little as possible, until the afternoon; a fruit-stone or pebble held around in the mouth, moderates thirst.

9. After any sort of exhausting effort, a cup of coffee, hot or cold, is an admirable sustainer of the strength, until nature begins to recover herself.

10. Unless after a long abstinence or great fatigue, do not eat very heartily just before a great undertaking; because the nervous power is irresistibly drawn to the stomach to manage the food eaten, thus drawing off that supply which the brain and muscles so much need.

11. If persons will drink brandy, it is incomparably safer to do so *after* an effort than before; for it can give only a transient strength, lasting but a few minutes; but as it can never be known how long any given effort is to be kept in continuance, and if longer than the few minutes, the body becomes more feeble than it would have been without the stimulus, it is clear that its use *before* an effort is always hazardous, and is always unwise.

12. Never go to sleep, especially after a great effort, even in hot weather, without some covering over you.

13. Under all circumstances, rather than lie down on the bare ground, lie in the hollow of two logs placed together, or across several smaller pieces of wood, laid side by side; or sit on your hat, leaning against a tree. A nap of ten or fifteen minutes in that position will refresh you more than an hour on the bare earth, with the additional advantage of perfect safety.

14. A *cut* is less dangerous than a bullet-wound, and heals more rapidly.

15. If from any wound the blood spirts out in jets, instead of a steady stream, you will die in a few minutes unless it is remedied; because an artery has been divided, and that takes the blood direct from the fountain of life. To stop this instantly, tie a handkerchief or other cloth very loosely BETWEEN!! the wound and the heart; put a stick, bayonet, or ramrod *between* the skin and the handkerchief, and twist it around until the bleeding ceases, and keep it thus until the surgeon arrives.

16. If the blood flows in a slow, regular stream, a vein has been pierced, and the handkerchief must be on the other side of the wound from the heart; that is, *below* the wound.

17. A bullet through the abdomen (belly or stomach) is more certainly fatal than if aimed at the head or heart; for in the latter cases the ball is often glanced off by the bone, or follows round it under the skin; but when it enters the stomach or bowels, from any direction, death is inevitable under almost all circumstances, but is scarcely ever instantaneous. Generally the person lives a day or two with perfect clearness of intellect, often *not* suffering greatly. The practical bearing of this statement in reference to the great future is clear.

18. Let the whole beard grow, but not longer than some three inches. This strengthens and thickens its growth, and thus makes a more perfect protection for the lungs against dust, and of the throat against winds and cold in winter, while in the summer a greater perspiration of the skin is induced, with an increase of evaporation; hence, greater coolness of the parts on the outside, while the throat is less feverish, thirsty, and dry.

19. Avoid fats and fat meats in summer and in all warm days.

20. Whenever possible, take a plunge into any lake or running stream every morning, as soon as you get up; if none at hand, endeavor to wash the body all over as soon as you leave your bed, for personal cleanliness acts like a charm against all diseases, always either warding them off altogether, or greatly mitigating their severity and shortening their duration. Let every sort of bath be completed within five minutes.

21. Keep the hair of the head closely cut, say within an inch and a half of the scalp in every part, repeated on the first of each month, and wash the whole scalp plentifully in cold water every morning.

22. Wear woolen stockings and easy-fitting, thick-soled shoes, keeping the toe and finger-nails always cut moderately close.

23. It is more important to wash the feet well every night, than to wash the face and hands of mornings; because it aids to keep the skin and nails soft, and to prevent chafings, blisters, and corns, all of which greatly interfere with a soldier's duty.

24. The most universally safe position, after all stunnings, hurts, and wounds, is that of being placed on the back, the head being elevated three or four inches only; aiding more than any one thing else can do, to equalize and restore the proper circulation of the blood.

25. The more weary you are after a march or other work, the more easily will you take cold, if you remain still after it is over, unless, the moment you cease motion, you throw a coat or blanket over your shoulders. This precaution should be taken in the warmest weather, especially if there is even a slight air stirring.

26. The greatest physical kindness you can show a severely-wounded comrade is first to place him on his back, and then run with all your might for some water to drink; not a second ought to be lost. If no vessel is at hand, take your hat; if no hat, off with your shirt, wring it out once, tie the arms in a knot, as also the lower end, thus making a bag, open at the neck only. A fleet person can convey a bucketful half a mile in this way. I've seen a dying man clutch at a single drop of water from the fingers' end, with the voraciousness of a famished tiger.

27. If wet to the skin by rain or by swimming rivers, keep in motion until the clothes are dried, and no harm will result.

28. Whenever it is possible, do, by all means, when you have to use water for cooking or drinking from ponds or sluggish streams, boil it well, and when cool, shake it, or stir it, so that the oxygen of the air shall get to it, which greatly improves it for drinking. This boiling arrests the process of fermentation which arises from the presence of organic and inorganic impurities, thus tending to prevent cholera and all bowel diseases. If there is no time for boiling, at least strain it through a cloth, even if you have to use a shirt or trowser-leg.

29. Twelve men are hit in battle, dressed in red, where there are only five, dressed in a bluish gray, a difference of more than two to one; green, seven; brown, six.

30. Water can be made almost ice cool in the hottest weather, by closely enveloping a filled canteen, or other vessel, with woolen cloth kept plentifully wetted and exposed.

31. While on a march, lie down the moment you halt for a rest; every minute spent in that position refreshes more than five minutes standing or loitering about.

32. A daily evacuation of the bowels is indispensable to bodily health, vigor, and endurance; this is promoted in many cases, by stirring a table-spoonful of corn (Indian) meal in a glass of water, and drinking it on rising in the morning.

33. Loose Bowels, namely, acting more than once a day, with a feeling of debility afterward, is the first step toward cholera; the best remedy is instant and perfect quietude of body, eating nothing but boiled rice with or without boiled milk; in more decided cases, a woolen flannel, fourteen feet long and fourteen inches wide, with two thicknesses in front, should be bound tightly around the abdomen, especially if marching is a necessity.

34. If the soldier takes his quota of hot coffee or a warm breakfast as soon as he gets up in the mornings, summer and winter, it will have a preventive influence against the general diseases of the camp, such as fevers, loose bowels, and bloody flux, which is incalculable; it is believed by eminent medical men that a rigid attention to this suggestion would diminish the army mortality from sickness at least thirty per cent

35. Whenever it is practicable, sleep with your feet to the camp-fire.

36. No soldier should at any time have less than two pair of woolen socks; those made by knitting-needles are greatly the best.

37. On a march have as little to carry as possible, every ounce becomes an appreciable burden in a half-day's tramp.

38. The instant you are burned or scalded place the part in cold water, this gives perfect relief in a second, then get some flour and cover the burned part completely, and let it remain until it gets well.

39. Thirst.—While on a march courageously resist thirst, especially in the early part of the day; for the more you drink the weaker will you become.

40. Water.—The East-Indians believe that they ward off the cholera which prevails in flat localities, where the water must be obtained from ponds, stagnant lakes, and sluggish streams, by boiling what is wanted for drinking purposes over night, and letting it stand in the open air until next morning, so as to reäbsorb the freshness, the oxygen, which the process of boiling had driven off. This is most especially needed in warm weather.

41. Blankets.—A small India-rubber blanket is a very great protection to the health, to lay on the ground or to throw over the shoulders during a storm, or on resting after getting into a heat, but unless *vulcanized* (if not, it becomes very sticky on being held close to the fire or in a very hot sun for a few moments) it has no endurance and is worthless.

42. Fatal forms of fever, loose bowels, and bloody discharges are often occasioned by a sudden check of perspiration from chilly winds or cold night air; so when perspiring even a little, either keep in moderate motion, go to a fire, or put on an additional coat or blanket.

43. Ardent Spirits.—It is beyond dispute, that always and every where, those who drink most of liquors in any shape, beer, brandy, whisky, or rum, soonest give out, soonest get sick, and are the slowest to recover. A very eminent English physician has lately communicated the fact, that out of one thousand members of the "Sick Clubs of Preston," who merely used, but did not abuse, spirituous liquors, twenty-three were laid aside by sickness every year for an average time of fifty-three days, while of an equal number who never touched liquor, there were only thirteen sick, averaging but twenty-three days; the number sick, the rapidity of recovery, the time lost, and the expense all being more than one half, or fifty per cent in favor of those who never used ardent spirits. Water quenches thirst better, if not very cold, especially if but a few swallows are taken at one time. Tea and coffee are better at meals for the soldier than water, but they should not be drank between meals; only in sips on a march, or under great exertions. The safest beverage in hot weather is molasses and water.

44. Eating.—Let it be at regular times as far as possible. That soldier is many-fold the safest from disablement, and a great variety of diseases, who will use three precautions in his eating: *First.* Let no bit of solid food go into the mouth larger than half the last joint of the little finger. *Second.* Chew it slowly and well. *Third.* Swallow not one atom between meals unless under very uncommon and urgent circumstances, so as to give the stomach time to rest, and gain strength to work up the succeeding meal. As often as possible, let there be at least five hours' interval between your eatings.

45. Flannel.—Wear it all over, in all weathers, except to use cotton drawers during the summer months, if you have them. Wash your flannels once a week if possible. When not, hang them up, also all your clothing, in the mid-day sun, whenever there is a chance to do so. Dry clothing is a great preservative of health. A single damp garment has sent many a person to the grave in a few days, and made others invalids for a lifetime.

46. Sleep as often and as much as you can; it is a great invigorator. Five minutes' sleep will refresh, invigorate, and strengthen more than any glass of liquor. It is better far to sleep too warm than even a little too cool.

47. The Three Plenties of pure air on high ground; of boiled or running water; and of bright-blazing fires, are the angels of health in any encampment.

48. Feet.—Thick-soled shoes, moderately loose, are best on a march, and it would be a great protection to the feet against chafings, etc., to rub a few drops of any kind of mild oil into the skin of the soles before a march.

49. A bullet-wound usually gives no external bleeding, and it is almost always safest to let it remain in the body, as it rarely does harm by so doing.

50. When a spent ball strikes the body, a wonderfully slight resistance turns it from its course, and prevents its being fatal—a bone, a tendon, or even a loose skin. A ball once entered under the chin, glanced around the neck, and came out near the place of entrance. A ball striking a rib has glanced off, and made half the circuit of the body. A ball once struck the abdomen in front, passed *around*, and came out at the back. A ball has pierced the skull-bone, but not having strength enough to enter the covering of the brain, which is of a tough, leathery nature, no serious harm was done.

51. The "wind" of a ball, as it is called sometimes, can not possibly kill a man.

52. A bullet in the body seldom does much harm. Great effort to get it out may make a case fatal which otherwise would have recovered.

53. A man need not necessarily die if a bullet lodges in the brain.

54. A bullet may pass entirely through the lungs without destroying life, as in the case of General Shields in the Mexican war, and he was living twelve years later. Lieutenant-General Winfield Scott received a ball in his shoulder at the battle of Molino del Rey, and it remained unextracted for several years.

55. Bullet wounds in the abdomen are nearly always fatal, while the majority of those in the chest recover.

56. A bullet may lodge in the heart itself without causing death, for several days, as in the case of Bill Poole, the pugilist.

57. BOWELS.—The very moment you experience any uncomfortable sensation about the bowels, bind around them tightly a piece of woolen cloth of any kind, to support them and keep them warm. It is a great remedy.

58. SUN AND AIR.—Keep in the open air as much as possible, but do not stand a moment in the hot sun if it can be avoided.

59. To have been "to the wars" is a life-long honor, increasing with advancing years, while to have died in the defense of your country, will be the boast and the glory of your children's children. "*Dulce et decorum est, pro patria mori.*"

